# Pioglitazone abrogates testicular damage induced by testicular torsion/detorsion in rats

**DOI:** 10.22038/ijbms.2019.33199.7929

**Published:** 2019-08

**Authors:** Nevertyty Mohamed Mahmoud, Soad Lotfy Kabil

**Affiliations:** 1 Department of Pharmacology, Faculty of Medicine, Zagazig University, Egypt

**Keywords:** Apoptosis, Inflammation, Ischemia, Pioglitazone, Reperfusion, Testis

## Abstract

**Objective(s):**

Testicular torsion/detorsion (T/D) is a well-known cause for infertility. Pioglitazone is an agonist of peroxisome proliferator activated receptor-gamma (PPAR-γ). Previous studies have shown that pioglitazone has anti-inflammatory, antioxidant and antiapoptotic properties. The present study hypothesized that pioglitazone may be protective against the testicular T/D tissue insults, and the possible pathophysiological mechanisms involved in this effect were also investigated.

**Materials and Methods:**

Rats were randomly divided into four groups: sham group, T/D group where testicular torsion was performed for 4 hr followed by 4 hr of detorsion and two pioglitazone-treated groups (1 mg/kg and 3 mg/kg, by single intraperitoneal injection 30 min prior to detorsion). At the end of reperfusion period, blood, ipsilateral and contralateral testicular tissue samples were obtained for biochemical and histopathological examination.

**Results:**

Pioglitazone reduced oxidative tissue damages, inflammatory mediators, and apoptotic markers and enhanced the total antioxidant status, and AMP-activated protein kinase level. Moreover, pioglitazone improved spermatogenesis evidenced by increased Johnsen’s score and reversed the histopathological damages induced by testicular T/D. The effects of pioglitazone were higher with the dose of 3 mg/kg.

**Conclusion:**

Pioglitazone exhibited a protective effect against the deleterious actions of testicular T/D. This beneficial potential of pioglitazone may be attributed to its antioxidant, anti-inflammatory and antiapoptotic properties, which was more obvious with the dose of 3 mg/kg. Pioglitazone may be a promising therapy for testicular T/D.

## Introduction

Torsion of spermatic cord is one of the most serious urological emergencies (1). Torsion - associated ischemia can lead to germ cell death. Although reperfusion is essential for survival of testicular cells, it is assumed to be responsible for bad prognosis, a phenomenon called ischemia - reperfusion (I/R) injury (2). Reperfusion is associated with over release of reactive oxygen species (ROS) (3), in addition to pro-inﬂammatory cytokines release including interleukin-6 (IL-6) and tumor necrosis factor- α (TNF- α), chemokines (monocyte chemoatractant protein-1 (MCP-1)) and cell adhesion molecules which results in recruitment of neutrophils and macrophages. ROS stimulate oxidative stress by oxidizing mitochondrial membranes, cell membrane lipids, proteins, and DNA, leading to cellular dysfunction, which is followed by germ cell apoptosis (4, 5). Sequel of spermatic cord torsion dose not only affect ipsilateral testis but also extends to contralateral testis. Pioglitazone is an agonist of peroxisome proliferator activated receptor-gamma (PPAR-γ), which is a member of nuclear hormone receptor superfamily. PPAR-γ regulates expression of genes involved in several physiological and pathological processes, such as glucose homeostasis, cellular differentiation, regulation of lipid metabolism, atherosclerosis, infertility, and inﬂammation (6). In a recent study, it was demonstrated that therapeutic effects of pioglitazone reach far beyond its use as an insulin sensitizer; several evidences revealed that pioglitazone attenuates I/R tissue insults in heart, brain and kidney (7). To the best of our knowledge, this study is the first to evaluate the possible protective potential of pioglitazone in testicular torsion-detorsion (T/D) injury. So, the current study hypothesized that pioglitazone may possess a protective effect against testicular T/D tissue damages, and the possible underlying mechanisms were investigated. 

## Materials and Methods

***Drugs and chemicals***

Pioglitazone and carboxy methylcellulose (1%, vehicle of pioglitazone) were purchased from Sigma- Aldrich. (St Louis, MO, USA). Ketamine (5%) was purchased from Sigma-Tec Pharmaceutical Industries (6^th^ of October City, Giza, Egypt). Xylazine (2%) was purchased from ADWIA Co. (10^th^ of Ramadan City, Egypt). 

***Animals***

Twenty-four adult male Wistar rats weighing 230–250 g were purchased from Faculty of Veterinary Medicine, Zagazig University. Rats were housed at the animal house, Faculty of Medicine, Zagazig University under standard laboratory conditions at a temperature of 22±2 ^°^C, relative humidity of 60%, 12-hr light-dark cycle and supplemented with commercial rodent chow and water *ad libitum*. Rats were acclimatized for 1 week before the experiment. The study was performed in Department of Pharmacology, Faculty of Medicine, Zagazig University. All experimental procedures were approved by the local Ethics Committee of Zagazig University, Egypt (IRB: 4183) and in accordance with National Institutes of Health guide for care and use of Laboratory animals (NIH Publications No. 8023, revised 1978). 

***Study design***

24 male Wistar rats were randomly allocated into 4 groups (6 rats each): sham group where rats subjected to surgical stress without testicular T/D and received carboxymethyl cellulose (1%) intraperitoneally (IP); testicular T/D group: rats were subjected to testicular torsion for 4 hr then testicular detorsion for another 4 hr and received carboxymethyl cellulose (1%) IP; pioglitazone group (Pio 1): rats treated with a single IP injection of pioglitazone 1 mg/kg (8) 30 min prior to detorsion; pioglitazone group (Pio 3): rats treated with a single IP injection of pioglitazone 3 mg/kg (8) 30 min prior to detorsion.

***Surgical procedure and tissue preparation***

Rats were anesthetized with IP injection of 50 mg/kg ketamine and 10 mg/kg Xylazine. Skin of scrotal area was shaved and prepared with 10 % povidone- iodine solution. A scrotal vertical midline incision was performed; torsion was induced by rotating right testis720° in a clockwise direction and maintained by fixing the testis in the scrotum with a 4-0 silk suture. Sham rats were subjected to the same surgical procedure without testicular rotation. After 4 hr torsion period, right testis was detorted and returned into the scrotum for 4 hr for reperfusion (9). At the end of the experiment, bilateral orchiectomies were performed. Blood samples were collected via cardiac puncture, centrifuged at 3000 g for 15 min to obtain a clear serum and was then stored at –20 ^°^C until use for biochemical assays. 

The excised testicular tissue (ipsilateral and contralateral) was washed with ice- cold saline and then longitudinally divided into two equal parts; one was fixed in 10% formalin for histopathological and immunohistochemical evaluation and the other was immersed immediately in liquid nitrogen and stored at -80 ^°^C. Testicular tissue samples were homogenized in cold potassium buffer phosphate solution. Homogenates were centrifuged for 15 min at 3000 g, and supernatants were used for biochemical assessment.

***Assay of testicular MDA***

Malondialdehyde (MDA) level in the testicular homogenate was quantified colorimetrically by using MDA assay kit purchased from MyBiosource Inc, San Deigo, USA and performed according to manufacturer’s instructions. 

***Assay of testicular GSH***

Reduced glutathione (GSH) content in the testicular homogenate was quantitatively assayed by the use of Sandwich ELISA kit (Catalog No. MBS046356, MyBiosource. Inc, San Deigo, USA) according to manufacturer’s guidelines.

***Assay of testicular SOD activity***

Testicular superoxide dismutase (SOD) activity evaluation was carried out according to a method (10) depending on ability of xanthine–xanthine oxidase system to inhibit nitro blue tetrazolium (NBT) reduction.

***Assay of pro-inflammatory markers in testicular homogenate***

Quantitative ELISA kits were used for determination of testicular TNF-α (ab100785) and MCP-1 (ab100778) purchased from Abcam, Cambridge, UK, according to manufacturer’s instructions.

***Assay for caspase -3 activity in testicular homogenate***

Caspase-3 activity was determined in testicular homogenate using apoptotic marker CASP3/Caspase 3 (Cat.no.LS-F11016, Life span Biosciences, USA) ELISA assay kit, according to manufacturer’s instructions.

***Assay of AMPK activity in testicular homogenate***

AMP- activated protein kinase (AMPK) activity was estimated in testicular homogenates using anti-AMPK antibody (ab80039) ELISA assay kit (Abcam, Cambridge, UK), according to manufacturer’s instructions.

***Assay of TOS in serum***

Serum total oxidant status (TOS) level was assayed using the method described by Erel (11) and were calculated in µmol H_2_O_2_ equivalent/l.

***Assay of TAS in serum***

Serum total antioxidant status (TAS) level was assayed according to the method described by Erel (12) and expressed as mmol Trolox equivalent/l.

***Histopathology***

The obtained testicular tissues were fixed in 10% formalin and embedded in paraffin blocks. 4-μm sections were obtained and stained with hematoxylin and eosin (H&E). Histopathological assessment of spermatogenesis was performed using Johnsen’s mean testicular biopsy score (MTBS) (13). MTBS was calculated by dividing sum of all scores by the total number of seminiferous tubules examined (Table 1). 

***Immunohistochemical detection of testicular IL-6 and Bcl-2***

4 μm thick testicular sections were deparaffinized, rehydrated in alcohol with serial rinsing in phosphate – buffered saline (PBS), and its endogenous peroxidase activity was quenched with 3 % H_2_O_2_ in methanol. For antigen retrieval, sections were pretreated in citrate buffer (pH 6.0) in a microwave. After that, the sections were incubated with rabbit polyclonal antibodies; anti Bcl-2 Ab (ab 194583) and anti-IL-6 Ab (ab 208113) specific for rat (Abcam, Cambridge, UK) at 4 ^°^C overnight. After washing the slides with PBS, sections were incubated at 37 ^°^C for 30 min with biotinylated polyvalent anti-rabbit antibody as a secondary antibody for primarily antibody detection, followed by incubation with streptavidin peroxidase, then stained with 3’3-diaminobenzedine plus chromogen for detection of peroxidase activity (D7679, Sigma, Aldrich) at room temperature for 10 min. Slides were counterstained with hematoxylin and examined under light microscope. Expression of IL-6 and Bcl-2 in testicular tissue was graded on scale depending on extent of brown stained cells (Scale 0 = no observed staining; scale 1= <10% of cells were stained weakly; scale 2= multifocal aggregates of uniformly stained cells, 10–75%; scale 3= diffuse positive staining). Histopathological analysis was performed blindly by an expert pathologist.

***Statistical analysis***

Analysis of data was performed using Graph Pad Prism software (v 6.0). Data were presented as mean ± standard error of mean (SEM). Multiple group comparisons were performed using one-way analysis of variance (ANOVA) followed by *post hoc* Tukey multiple comparisons test. Probability values,* P*<0.05 were considered as statistically significant. 

## Results

***Effect of pioglitazone on testicular oxidant and antioxidant status***

**Figure 1:**
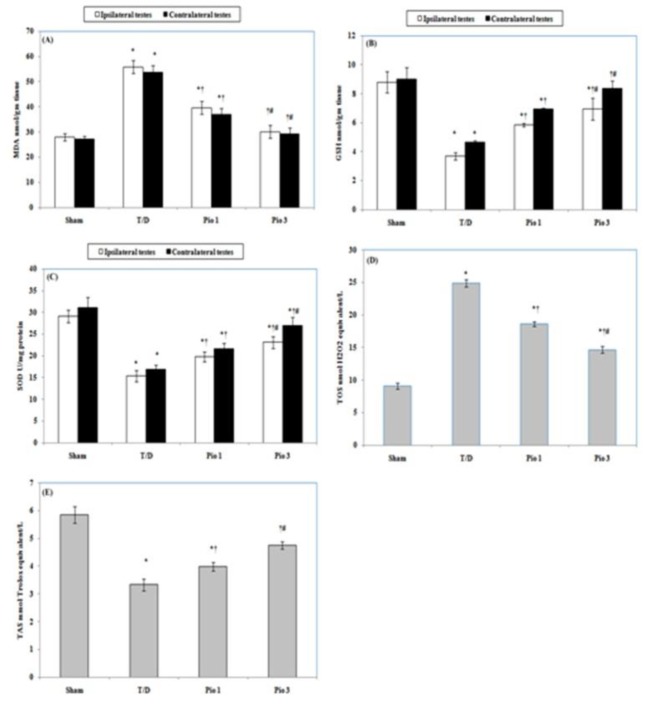
Effect of testicular torsion/detorsion(T/D) alone or with pioglitazone (Pio) (1 and 3 mg/kg) on (A) malondialdehyde (MDA), (B) reduced glutathione (GSH), and (C) superoxide dismutase (SOD) activity in ipsilateral and contralateral testes, (D) serum total oxidant status (TOS) and (E) serum total antioxidant status (TAS). Data are means±SEM. * compared to sham group, † compared to T/D group and # compared to Pio 1 group (*P*<0.05)

Tissue MDA levels were significantly (*P*<0.05) increased in both ipsilateral and contralateral testes of T/D group by 100.25% and 92%, respectively, compared to those of sham group. Pioglitazone (1 mg/kg) treatment (Pio 1 group) significantly (*P*<0.05) reduced tissue MDA levels in ipsilateral testes by 29.1 % and in contralateral testes by 31.2 % compared to T/D group. Further significant (*P*<0.05) reductions in MDA levels by 46.2% in ipsilateral and 46% in contralateral testes were detected in pioglitazone 3 mg/kg (Pio 3)-treated group (Figure 1A). On contrary, testicular GSH levels and SOD activity in T/D ipsilateral testes were decreased significantly (*P*<0.05) by 58% and 47.2%, respectively. Also, pioglitazone 3 mg/kg in contralateral testes attenuated GSH levels and SOD activity by 48.6% and 45.7%, respectively compared to sham rats. Pio 1 group showed significant (*P*<0.05) elevation in GSH levels by 58.3% and 47.2% and SOD activity by 29% and 38.3% in ipsilateral and contralateral testes, respectively in relation to T/D group. Additionally, the testicular GSH levels showed significant (*P*<0.05) increase by 88% and 81% and SOD activity by 51% and 72.1%, in pio 3 group in ipsilateral and contralateral testes, respectively compared to T/D group (Figure 1B,C). 

***Effect of pioglitazone on serum TOS and TAS***

Serum TOS level in T/D group was significantly (*P* <0.05) increased by 174% compared to sham group. Pioglitazone at both doses of 1 mg/kg and 3 mg/kg showed significant (*P*<0.05) reduction in serum TOS level by 25.2% and 41.1%, respectively in respect to T/D group (Figure 1D). Compared to sham group, serum TAS level was significantly (*P*<0.05) decreased by 43.2% in T/D group. Pioglitazone treated groups at doses of 1 mg/kg and 3 mg/kg showed significant (*P*<0.05) increase in the serum TAS level by 19.5 % and 43%, respectively compared to T/D group (Figure 1E).

***Effect of pioglitazone on testicular pro-inflammatory mediators***

T/D group showed significant (*P*<0.05) increase in TNF- α by 235% and 197% and MCP-1 levels by 143.5% and 132 % in ipsilateral and contralateral testes, respectively compared to sham rats. Pio 1 group showed significant (*P*<0.05) decrease in the levels of TNF- α by 35.3% and 34% and MCP-1by 31 % and 28.6 % in ipsilateral and contralateral testis, respectively compared to T/D group. More reductions in these inflammatory mediators levels were encountered in Pio 3 rats as evidenced by significant (*P*<0.05) decrease in TNF- α by 48.6%, and 47% and MCP-1 by 45% and 46.3% in ipsilateral and contralateral testes, respectively (Table 2)**. **

***Effect of pioglitazone on testicular level of AMPK***

Testicular T/D produced significant (*P*<0.05) increase in AMPK level by 41% in ipsilateral and 42.5% in contralateral testes compared to sham group. AMPK levels were significantly (*P*<0.05) decreased in Pio 1 and Pio 3 groups by 19.4% and 43% in ipsilateral testis and by 24% and 46% in contralateral testes, respectively, compared to testicular T/D group (Table 2). 

***Effect of pioglitazone on testicular caspase -3 activity***

T/D group showed significant (*P*<0.05) increase in caspase -3 activity by 181.5% and 163% in ipsilateral and contralateral testis, respectively in relation to sham rats. Caspase -3 activity was significantly (*P*<0.05) decreased in Pio 1 and Pio 3 groups by 30.6%, and 58 % in ipsilateral testis and by 29.4% and 58.4% in contralateral testes, respectively in comparison with T/D group (Table 2). 

***Histopathological analysis for assessment of spermatogenesis***

**Figure 2:**
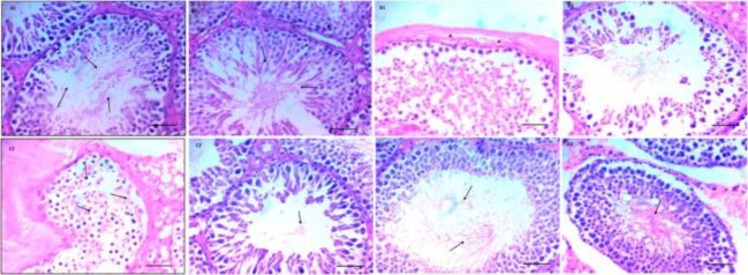
Photomicrographs of (H & E X 400, scale bar represent 50 µm) testicular sections. (A1 and A2) Testicular tissue of ipsilateral and contralateral testes of sham rat represents normal seminiferous tubule containing normal layers of spermatogenic cells up to mature sperms (arrows) and surrounded by normal basement membrane (arrowheads). (B1 and B2) Ipsilateral and contralateral testes of rat exposed to torsion / detorsion show marked sloughing and disorganization of spermatogenic cells, thickening of basement membrane (arrowheads) and absence of mature sperms. (C1 and C2) Demonstrate mild reductions of spermatogenic cells layers with few sperms in lumen (arrows) of ipsilateral and contralateral testes of rat exposed to torsion / detorsion and treated with pioglitazone 1 mg/kg. (D1 and D2) represent normal layers of spermatogenic cells with mature sperms in lumen (arrows) and normal thicknesses of basement membrane (arrowheads) in ipsilateral and contralateral testes of rat exposed to torsion / detorsion and treated with pioglitazone 3 mg/kg

Rats in sham group displayed normal testicular architecture of normal seminiferous tubules, multiple normal layers of spermatogenic cells from spermatogonium up to mature sperms and surrounded by normal basement membrane, while testicular tissues of T/D group exhibited sloughing, disorganization of spermatogenic cells with absence of mature sperms and thickening of basement membrane of both ipsilateral and contralateral testes. Treatment with pioglitazone 1 and 3 mg/ kg showed significant dose-dependent increase in layers of spermatogenic cells with mature sperms in lumen of both ipsilateral and contralateral testes (Figure 2). While significant increase in Johnsen’s score was observed in Pio 1 group, further significant increments in the score were detected in Pio 3 group affecting both ipsilateral and contralateral testis (Table 2).

***Immunohistochemical testicular Bcl-2 expression***

**Figure 3:**
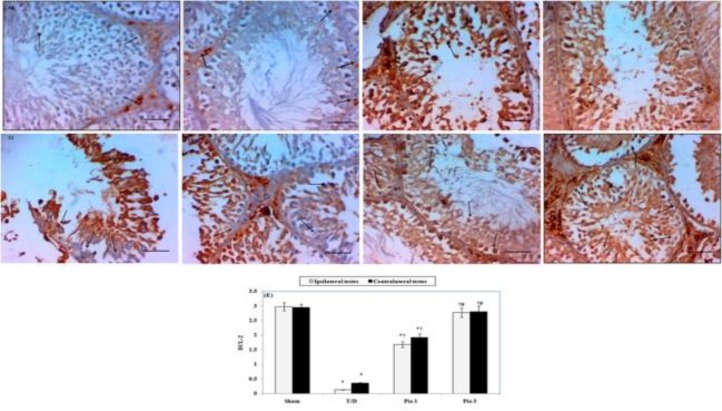
A-D Photomicrographs represent immunohistochemical staining of Bcl-2 in testicular tissues of different groups (magnification × 400, scale bar represent 50 µm). (A 1) ipsilateral testis and (A 2) contralateral testis sections of sham rat show diffuse cytoplasmic staining of Bcl- 2 (arrows). Representative section from ipsilateral (B 1) and contralateral (B 2) testes of rat exposed to torsion / detorsion (T/D) show less Bcl- 2 staining (arrows). (C 1 and 2) Representative sections of ipsilateral and contralateral testes of rat exposed to T/D and treated with pioglitazone 1 mg /kg (Pio 1) show moderate expression of Bcl -2(arrows). Sections of ipsilateral (D 1) and contralateral (D2) testes of rats exposed to T/D and treated with 3 mg/kg pioglitazone (Pio 3) show increased Bcl- 2 cytoplasmic staining (arrows). (E) Bar histogram demonstrating scores of Bcl-2 expression. Results shown are means ± SEM of six rats/ group. * compared to sham group, † compared to T/D group and # compared to Pio 1 group (*P*<0.05)

Immunohistochemical staining of testicular tissue revealed expression of Bcl-2 in sham rats (Figure 3(A)). Testicular T/D group showed significant (*P* <0.05) decrease in Bcl-2 expression in both ipsilateral and contralateral testes compared to sham group (Figure 3(B)). Pio 1 and Pio 3 treated groups showed significant (*P*<0.05) increase in Bcl-2 expression in respect to testicular T/D group (Figure 3(C), (D), (E)).

***Immunohistochemical testicular IL-6 expression***

**Figure 4:**
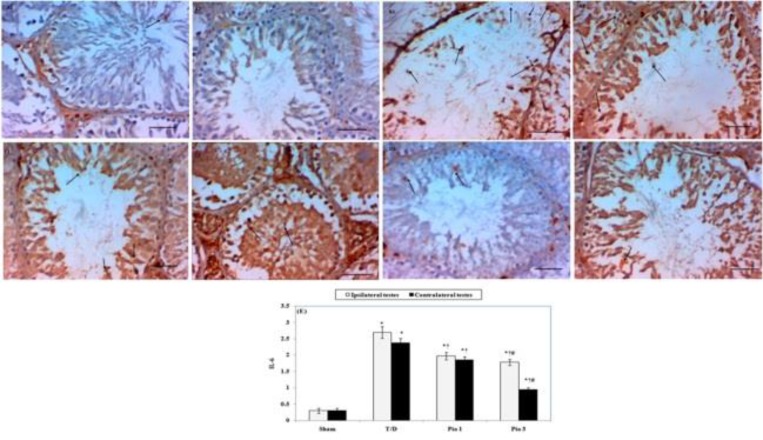
A-D Photomicrographs represent immunohistochemical staining of interleukin 6 (IL-6) in testicular tissues of different groups (magnification × 400, scale bar represent 50 µm). Ipsilateral (A1) and Contralateral (A2) testes of sham rat show weak cytoplasmic expression of IL-6 (arrows). Rat exposed to testicular torsion / detorsion (T/D) show marked cytoplasmic expression of IL-6 (arrows) in both ipsilateral (B1) and contralateral (B2) testes. Pioglitazone treatment (1 and 3 mg/kg) produced dose-dependent decrease in IL-6 expression (arrows) in ipsilateral (C1 and D1) and contralateral testes (C2 and D2). (E) Bar histogram represents scores of IL-6 expression. Data are means±SEM. * compared to sham group, † compared to T/D group and # compared to Pio 1 group (*P*<0.05)

Testicular T/D group showed positive staining for IL-6 in ipsilateral and contralateral testicular tissue (Figure 4(B)) compared to sham group (Figure 4(A)). Pio 1 and Pio 3 groups showed negative staining for IL-6 compared to testicular T/D group (Figure 4C, D, E).

## Discussion

The current study demonstrates for the first time a protective potential for pioglitazone in the testicular injury induced by unilateral T/D. Being end arteries, testicular arteries make the testes especially susceptible to ischemic injury. In agreement with previous studies (14,15), we reported that unilateral testicular rotation for four hours followed by four hours reperfusion caused significant testicular damage in both ipsilateral and contralateral testes, as evidenced by biochemical (increased levels of MDA, MCP- 1, TNF- α, caspase-3, AMPK, TOS and decreased levels of GSH, SOD, TAS), immunohistochemical (increased IL-6 and decreased Bcl -2) and histopathological analyses, which revealed decreased Johnsen’s score of spermatogenesis. To explain contralateral testicular injury, multiple theories were implicated including autoimmunization against the spermatogonia, decrease in testicular blood flow caused by a reflex sympathetic response and ROS generation after detorsion (15). In the present study, pretreatment with pioglitazone (1 and 3 mg/kg) significantly decreased the levels of MDA, MCP- 1, TNF- α, caspase-3, AMPK, TOS, and IL-6 and increased the levels of GSH, SOD, TAS and Bcl -2. These beneficial effects of pioglitazone are supported by increase in Johnsen’s score of spermatogenesis. Based on the literature argument, oxidative stress plays a central role in pathophysiology of testicular T/D-induced injury. Unfortunately, both phases of torsion and detorsion result in burst of mitochondrial ROS generation, which consumes natural antioxidants, leading to oxidative stress. The liberated ROS exert its deleterious effects by triggering oxidative damage to lipids, proteins and DNA (16). Consistent with earlier studies (15, 17), the current study demonstrated increased MDA in both ipsilateral and contralateral testicular tissues as indicator of lipid peroxidation. In the present work, pioglitazone decreased MDA level in both ipsilateral and contralateral testes. In the same context, previously published studies confirmed inhibition of lipid peroxidation by pioglitazone supplementation (18, 19). To declare potential mechanisms by which pioglitazone abrogated T/D induced - oxidative stress, we assayed the effects of pioglitazone on antioxidant system in both ipsilateral and contralateral testes. Testicular level of GSH, an important scavenger of ROS, was decreased bilateral in rats exposed to T/D, while administration of pioglitazone elevated GSH levels in a dose-dependent manner. The current results are in line with a previous study (20) that reported enhancement of GSH levels in rats exposed to renal I/R and pretreated with pioglitazone. SOD is a vital antioxidant that catalyzes partitioning of superoxide radicals into hydrogen peroxide or molecular oxygen to be scavenged by GSH. Previous studies have shown that SOD activity is reduced due to testicular T/D (21). In the present study, we demonstrated decline in SOD activity in both testes of T/D rats, meanwhile pioglitazone maintained SOD activity that this effect can be attributed to its antioxidant property. In harmony with that, pioglitazone increased SOD activity and protected against paracetamol-induced hepatotoxicity (22). This eminent antioxidant potential of pioglitazone is additionally confirmed in this study by decreasing the serum TOS and increasing the TAS. Pioglitazone also increased the TAS in diabetic rabbits (23). These data attribute the rapid and direct inhibition of complex I (via the attenuation of NADPH oxidase activity) and complex III of mitochondrial respiration to antioxidant effect of pioglitazone, which leads to decrease in generation of ROS from mitochondria (24-26). Collectively, we concluded that the antioxidant potential of pioglitazone is due to inhibition of ROS generation and bolstering testicular antioxidant parameters including GSH and SOD.

**Table1:** Histological criteria and Johnsen’s score for spermatogenesis assessment

Score	Characters
Score–10	Complete spermatogenesis with many spermatozoa present
Score–9	Slightly impaired spermatogenesis with many late spermatids, disorganized epithelium
Score–8	Less than five spermatozoa per tubule, few late spermatids
Score–7	No spermatozoa, no late spermatids, many early spermatids
Score–6	No spermatozoa, no late spermatids, few early spermatids
Score–5	No spermatozoa or spermatids, many spermatocytes
Score–4	No spermatozoa or spermatids, few spermatocytes
Score–3	Spermatogonia only
Score–2	No germinal cells, Sertoli cells only
Score–1	No seminiferous epithelium

**Table 2:** Effect of pioglitazone 1 and 3 mg/kg on testicular inflammatory markers, caspase – 3 activity and Johnsen’s score in rats exposed to torsion/ detortion

Parameters	Sham	T/D	Pio 1	Pio 3
TNF- α (pg/g tissue)				
I C	11.43 ± 0.07 11.15± 0.04	38.29 ± 2.11* 33.16 ± 2.03*	24.76 ± 1.93*† 21.87± 1.56*†	19.67 ± 0.94*†# 17.57 ± 0.84*†#
MCP-1(ng/ml)				
I C	24.54 ± 1.08 23.94 ± 1.07	59.77±1.62* 55.52 ± 1.43*	41.19 ± 1.44 *† 39.65 ± 1.38*†	32.94 ± 1.54*†# 29.79 ± 1.84*†#
AMPK (ng/mg tissue)				
I C	23.71±1.35 23.95± 1.21	33.46± 1.75* 34.12± 1.96*	39.95± 2.3 *† 42.23± 2.52*†	47.85±3.47*†# 49.78±3 .1*†#
Caspase –3 (ng/g tissue)				
I C	6.8±0.41 6.8±0.34	19.14±1.31* 17.89 ± 1.26*	13.29±1.25*† 12.63±1.18*†	8.04±0.91*†# 7.45±0.73*†#
Johnsen’s score				
I C	9.56± 0.41 9.57 ± 0.43	4.17± 0.25 * 4.33 ± 0.28*	6.94± 0.27* † 7.17± 0.30*†	7.85± 0.32 *†# 8.13± 0.38*†#

Values are expressed as mean± SEM (n=6).I= ipsilateral, C= contralateral. Pio: pioglitazone; TNF-α: tumor necrosis factor alpha; MCP-1: monocyte chemoattractant-1; AMPK: AMP- activated protein kinase

* significantly different from sham group (P<0.05),

† significantly different from T/D group (P <0.05),

# significantly different from Pio 1 group (P<0.05).

In the current study, pioglitazone ameliorated the increased levels of TNF–α and IL-6 in both ipsilateral and contralateral testes after unilateral T/D. A PPAR-γ dependent inhibition of TNF–α and IL-6 was reported in previous studies against carrageenan-induced inflammation (27), forebrain I/R injury (28) and myocardial I/R injury (29).

In this study, pioglitazone decreased MCP-1 level in testicular tissue. MCP-1 is a principle chemokine for recruitment of monocytes into testicular interstitium to be matured into resident – macrophages (30), which can further contribute to more release of inflammatory mediators and ROS. Therefore, pioglitazone–induced inhibition of MCP-1 provides indirect antioxidant mechanism for attenuating the release of ROS. Testicular T/D injury is associated with increased MCP-1 expression by leydig cells, macrophages, peritubular cells and perivascular cells (31). ITO and co-workers (32) demonstrated tendency of pioglitazone to reduce myocardial infarct size resulted from myocardial I/R through attenuating expression of MCP-1. In the same line, pioglitazone was established to produce anti-inflammatory effect against cecal ligation and puncture-induced sepsis in mice through attenuating the expression of MCP-1 and IL-6 (33). Anti-inflammatory activity of pioglitazone is approved to be independent from its antidiabetic action (34). 

Apoptosis is an essential physiologic process that takes place during normal spermatogenesis; however, overproduction of ROS initiates intrinsic apoptotic pathway through increasing the expression of pro-apoptotic Bax and decreasing anti-apoptotic Bcl-2 with subsequent destabilization of mitochondrial membrane (35). Membrane destabilization leads to release of mitochondrial cytochrome-C into the cytoplasm, and activation of cascade of caspases including caspase-3, which eventually activates caspase-activated DNase and degrades DNA (36). Additionally, TNF-α stimulates its receptors on mitochondrial membrane to activate extrinsic pathway of apoptosis (37); these pathways result in germ cell specific apoptosis. The present study detected that pioglitazone increased expression of anti- apoptotic Bcl -2 and decreased the level of caspase-3 in testicular tissue of rotated and non- rotated testicles that can be suggested as an anti- apoptotic mechanism of pioglitazone.

In agreement with our results, Hu *et al.* (38) illustrated renoprotective effect of pioglitazone against I/R injury and attributed this effect to enhancement of Bcl-2 expression, which antagonizes death-promoting activity of Bax, so mitochondrial activity and cell integrity are maintained. 

The current results demonstrated an increase in AMPK activity in T/D rats, which indicates presence of innate mechanism to counteract testicular injury, and pioglitazone treatment was associated with more increase in AMPK level. Previous studies detected an increase in AMPK levels in myocardial (39), hepatic (40) and renal (41) I/R injury. AMPK is considered as energy sensor; it is phosphorylated and activated by increased AMP/ATP ratio during tissue ischemia. Oxidative stress can also activate AMPK. The protective effect of AMPK against many types of I/R injury is well- established. This protection might be due to improving metabolic stress by maintaining metabolic balance through activating catabolic (ATP generating) pathways and attenuating anabolic (ATP consuming) steps (42). The anti-inflammatory role of AMPK was also investigated, and the results showed that AMPK activators suppress the release of TNF- α and IL -6 from macrophage in response to lipopolysaccharide; additionally, activation of AMPK decreased the expression of adhesion molecules, infiltration of leukocytes and decreased the level of pro-inflammatory cytokines in hepatic I/R injury (40). Moreover, the antiapoptotic effect of AMPK against hepatic I/R injury in rats was detected (43). The antioxidant character of AMPK was reported to be a result of increasing level of SOD, and catalase (44) and inhibiting the release of ROS through inhibition of NAD(P)H oxidase (45) .Many researches proved the ability of pioglitazone to augment the level of AMPK, and this may participate in protection against atherosclerosis (46), cisplatin-induced nephrotoxicity (47) and myocardial injury caused by metabolic syndrome (48).

Additionally, pioglitazone reduced congestion, sloughing and disorganization of spermatogenic cells and increased the layers of spermatogenic cells with mature sperms in the lumen with subsequent increase in the Johnsen’s score. The aforementioned histopathological results supported the biochemical and immunohistochemical results. Avlan *et al.* (9) reported similar histopathological derangements of testis exposed to testicular T/D. 

## Conclusion

The present study revealed that unilateral testicular torsion caused bilateral testicular damage with increased oxidative, inflammatory and apoptotic markers. Pioglitazone reversed testicular histopathological derangements, reduced inflammation, and enhanced antioxidative status and antiapoptotic markers. These effects of pioglitazone are dose-dependent. So, the present study demonstrated the protective effect of pioglitazone against testicular T/D, which was attributed to its antioxidant, anti-inflammatory and antiapoptotic properties. 
